# Supplementary material for: The transcription factor DDIT3 is a potential driver of dyserythropoiesis in myelodysplastic syndromes
Source: Nat Commun. 2022 Dec 9;13:7619. doi: 10.1038/s41467-022-35192-7 (PMC9734135; doi:10.1038/s41467-022-35192-7)
Supplement: Supplementary file 1 — Supplementary information [file 41467_2022_35192_MOESM1_ESM.pdf]

The transcription factor DDIT3 is a Potential Driver of Dyserythropoiesis in Myelodysplastic Syndromes

SUPPLEMENTARY INFORMATION

SUPPLEMENTARY FIGURE LEGENDS

Figure S1. Differential gene expression characterizes HSCs from young and older healthy adults, and MDS patients. (a) Flow cytometry strategy used for the isolation of HSCs ($CD34^{+}$ $CD38^{-}$ $CD90^{+}$ $CD45RA^{-}$) and $CD34^{+}$ cells. (b) Bar-plot representing the number of differentially expressed genes (DEG) in HSCs in the comparisons: healthy young versus older adults (aging), healthy older adults versus MDS (transition to the disease) and young adults versus MDS (the more distant stages). Up- and down-regulated genes are indicated in orange and blue, respectively. Source data are provided as a Source Data file.

Figure S2. Different transcriptional dynamisms of human HSCs in the aging-MDS axis associate with specific biological functions. (a) Bubble plot representing statistically significant biological processes and pathways enriched in genes specifically altered in aging (C1: red dots; C2: blue dots). Bubble size depicts -log10(p-value) and x axis represents GeneRatio. (b-d) Examples of genes from C1 involved in the modulation of immune response and activation of the NF-Kß pathway (b), and from C2 related to DNA damage (c) and cell proliferation (d). The normalized expression in young healthy adults (blue, young_HA), older healthy adults (orange, older_HA) and MDS (red) patients is depicted. N=37 biologically independent samples (n=17 young adults, 8 older adults, 12 MDS patients). Each point represents a donor or patient and the mean +/- SD is shown. (e) Bubble plot representing statistically significant biological processes and pathways enriched in genes showing a continuous deregulation in the axis (C5: red dots; C6: blue dots). Log_{10}(p-value) and GeneRatio are depicted as in (a). (f-h) Examples of genes from C6 involved in DNA repair (f) or transcriptional regulation (g), and from C5 representing negative cell cycle regulators (*TSPYL2* and *TGB1*) and extracellular matrix organization

factors (*COL9A3* and *ELN*) (h) are represented. Representation of gene expression, and number of biologically independent samples as in (b). (i) Heatmap showing the statistical significance (Log_{10}p-value) of enrichment of the genes of C2 and C6 in biological processes related to DNA replication, cell proliferation and DNA damage repair as determined by GO analysis. Modified Fisher exact test was used to calculate p-values. (j) Examples of genes from C8 involved in protein or RNA metabolism are represented. Representation of gene expression, and number of biologically independent samples as in (b). (k) Normalized expression of several genes from C4 involved in DNA repair (*SETX*), DNA replication (*REV3L*), proliferation (*GRB2*) and adhesion (*RAC2*). Representation of gene expression, and number of biologically independent samples as in (b). (l) Normalized expression of genes from C3 involved in miRNA processing and IFN production. Representation of gene expression, and number of biologically independent samples as in (b). Source data are provided as a Source Data file.

Figure S3. *DDIT3*-overexpression alters the transcriptional profile of healthy HSCs. (a) Dot plot showing the normalized expression of *DDIT3* in MDS patients with or without *DDIT3* overexpression. Each dot represents one individual the mean +/- SD is depicted. N=12 biologically independent samples (n=6 *DDIT3*-low and n=6 *DDiT3*-high expressing MDS patients). The statistical significance of the differential expression between both groups is shown and was calculated using Mann Whitney U test. (b) Normalized expression of *DDIT3* in previously published datasets GSEA114922 and GSE19429, which analyzed $CD34^+$ cells from control and MDS samples cases using RNA-seq and expression arrays, respectively. GSEA114922: n=90 biologically independent samples (82 MDS patients and 8 healthy older adult samples). GSE19429: n=200 biologically independent samples (183 MDS patients and 17 healthy individuals). Data are presented as mean values +/- SD. (c) Immunofluorescence with an anti-DDIT3 antibody of healthy CD34+ cells transduced with a control or a *DDIT3*-overexpressing plasmid. Bars indicate scale: 50 μm. These experiments were repeated three independent times with similar results. (d) K562 cells were transduced with the overexpression system, and extracts were obtained at 8 days and immunoblotted with the indicated antibodies. These experiments were repeated three independent times with similar results. (e-g) Expression of genes involved in DNA repair (e), adhesion (f), and heme metabolism (g)

in *DDIT3*-overexpressing HSCs and in control cells. Expression is presented as fold change over control cells for two independent biological replicates. (h) GSEA plots depicting the enrichment upon *DDIT3* overexpression in gene signatures representing genes up- and down-regulated in HSCs from MDS patients when compared to those from older healthy adults. The NES and adjusted p-values are indicated for each signature. Source data are provided as a Source Data file.

Figure S4. *DDIT3* overexpression impairs normal erythroid differentiation. (a) Micrographs of representative BFU-E colonies formed by healthy $CD34^+$ cells transduced with a control or a *DDIT3*-overexpressing plasmid. Bars indicate scale: 500 μm. Experiments were repeated three independent times with similar results. (b) Box-plots (center line, median; box limits, 25th and 75th percentiles; whiskers, min to max) representing the percentage of $CD11b^+$, $CD14^+$ or $CD15^+$ cells at 10 and 14 days of granulocytic differentiation. Three biological replicates are depicted; ns: no statistically significant differences. (c) Cumulative number of cells at 0 and 14 days for control and *DDIT3*-overexpressing cells. (d) Bar and scatter plot representing the percentage of cells in different phases of the cell cycle for control and *DDIT3*-overexpressing cells. (e) Bar and scatter plot depicting the percentage of cells stained with Annexin V and/or DAPI for control and *DDIT3*-overexpressing cells. Panels c-e represent the average +/- SD of three independent experiments. Statistically significant differences are indicated; ns indicates no statistically significant differences. (f) Left: flow cytometry charts representing advanced erythroid differentiation (stages I-IV) of HSCs transduced with a control or a *DDIT3*-overexpressing plasmid at day 14 of differentiation. Right: bar and scatter plots representing the percentage of cells observed for stages I-IV. Average +/- SD of three independent experiments is depicted; statistically significant differences are indicated. (b-f) P-values were calculated with multiple t test corrected for multiple comparisons using the Holm-Sidak method. (g) UMAP plot of the transcriptome of control and *DDIT3*-overexpressing cells subjected to ex vivo differentiation for 14 days. (h) UMAP plots depicting the number of counts (left), the percentage of ribosomal RNA (center), and the normalized expression of ATG3 (right) of the erythroid clusters. (i) Heatmap representing the prediction score for each of the cells in figure 4E whose identity was assigned based on transcriptional data from a previous work. (j) Violin plot

representing the log normalized expression of *DDIT3* in each of the erythroid states of figure 4E, for control (blue) and *DDIT3*-overexpressing cells (red). Statistically significant p-values, calculated with Model-based Analysis of Single-cell Transcriptomics, are indicated. Source data are provided as a Source Data file.

Figure S5. *DDIT3* overexpression prevents normal activation of transcriptional programs guiding erythroid differentiation. (a) Heatmap representing the z-score of DEGs between control cells and cells overexpressing *DDIT3* at day 14. (b-c) Normalized expression of erythroid differentiation (b) and stem cell genes (c) in $CD34^+$ cells at different time points of erythroid differentiation. N=2 biologically independent samples. (d-e) Gene expression trends of early hematopoietic progenitor genes (d) and erythroid differentiation factors and hemoglobin genes (bottom) calculated by pseudotime are represented as a smooth fit (prediction of the linear model) with the SD of the fit shown in a lighter shade for control (blue) and *DDIT3*-overexpressing cells (red). P-values showing the statistical differences between both trends of expression are indicated, and were calculated using the Wilcoxon test. (f) Ridge plot showing AUC scores for several regulons showing increased activity upon *DDIT3* upregulation (*JUND, ARID3A, ARID1A*) in control (blue) and *DDIT3*-overexpressing cells (pink) at different stages of erythroid differentiation. (g) Gene expression trends calculated by pseudotime of TFs guiding regulons showing decrease activity in *DDIT3*-overexpressing cells, are represented as a smooth fit with the standard deviation of the fit shown in a lighter shade for control (blue) and *DDIT3*-overexpressing cells (red). P-values showing the statistical differences between both trends of expression are indicated, and were calculated using the Wilcoxon test. (h) Immunoblot for CEBPB (different isoforms indicated) and DDIT3 after the immunoprecipitation with an anti-DDIT3 antibody or an IgG control in cells transduced with a control or a *DDIT3*-overexpressing plasmid. These experiments were repeated three independent times with similar results. (i) Immunoblot for CEBPG and DDIT3 after the immunoprecipitation with an anti-CEBPG antibody or an IgG control in cells transduced with a control or a *DDIT3*-overexpressing plasmid. These experiments were repeated three independent times with similar results. (j) Ridge plot showing AUC scores for regulons of *CEBPG* and *CEBPB* in control (blue) and *DDIT3*-overexpressing cells

(pink) at different stages of erythroid differentiation. Source data are provided as a Source Data file.

Figure S6. *DDIT3* knockdown in $CD34^+$ cells from MDS patients showing anemia restores erythroid differentiation. (a) MDS CD34+ cells were transduced with the knockdown system, and after 3 days, infected cells (GFP+) were FACS-isolated and immunofluorescence was performed with an anti-DDIT3 antibody. Bars indicate scale: 50 μm. These experiments were repeated three independent times with similar results. (b) K562 cells were transduced with the knockdown system, and extracts were obtained at 2 days and immunoblotted with the indicated antibodies. These experiments were repeated three independent times with similar results. (c) Left: flow cytometry charts representing advanced erythroid differentiation (CD71 and CD235a markers; stages I-IV) of CD34+ cells 5 MDS cases at days 7 and 14 of differentiation. Right: bar-plots representing the percentage of cells observed for stages I-IV. (d) Normalized expression of *DDIT3* in patient MDS13 upon transduction of $CD34^+$ cells with the indicated shRNAs, culture in the myeloid-erythroid differentiation system for 7 and 14days, and FACS-isolation of transduced cells. (e) Normalized expression of *DDIT3* in patient MDS14 upon transduction of $CD34^+$ cells with the indicated shRNAs, culture in the myeloid-erythroid differentiation system for 7 days, and FACS-isolation of transduced cells. (f-g) Left: flow cytometry charts representing advanced erythroid differentiation (CD71 and CD235a markers; stages I-IV) for cells from patient MDS14 (f) and MDS15 (g) harboring a control shRNA (shCtrl) or shRNAs targeting *DDIT3*, after ex vivo myeloid differentiation in liquid culture. Right: bar-plots representing the percentage of cells observed in stages I-IV. (h) Normalized expression of *DDIT3* in healthy young adults (blue), healthy older adults (orange) and MDS (red) samples used for the knockdown experiments. N=30 biologically independent samples (17 young adults, 8 older adults, and 5 MDS patients). Each point represents a donor or patient and the mean +/- standard deviation (SD) is shown for each group. Each MDS patient is indicated with its sample ID (Table S2 of the manuscript). Source data are provided as a Source Data file.

Figure S7. *DDIT3* knockdown and overexpression in $CD34^+$ cells from MDS patients renders opposite effects on erythroid differentiation. (a-b) Left: flow cytometry charts

representing advanced erythroid differentiation (CD71 and CD235a markers; stages I-IV) for cells from patients MDS16 (a) and MDS17 (b) harboring a control shRNA (shCtrl) or shRNAs targeting *DDIT3*, after ex vivo myeloid differentiation in liquid culture. Right: bar-plots representing the percentage of cells observed in stages I-IV. (c) Left: flow cytometry charts representing advanced erythroid differentiation (CD71 and CD235a markers; stages I-IV) of CD34+ cells from patients MDS16 and MDS17 showing low basal levels of *DDIT3* expression, transduced with a control or *DDIT3*-overexpression plasmid and differentiated for 14 days. Right: bar-plots representing the percentage of cells observed on the left. (d-e) Normalized expression of hemoglobins (d) and stem cell genes (e) of $CD34^{+}$ cells from patient MDS14 transduced with a shRNA control or and shRNA targeting *DDIT3* and subjected to 7 days of ex vivo differentiation. (f) Heatmap of z-scores of genes characteristic of proerythroblasts, early and late basophilic erythroblasts (left), and of genes expressed in poly- and orthochromatic stages (right), for cells from patient MDS14 transduced with a shRNA control, or an shRNA targeting *DDIT3* and subjected to 7 of ex vivo differentiation. Source data are provided as a Source Data file.

Figure S1

a

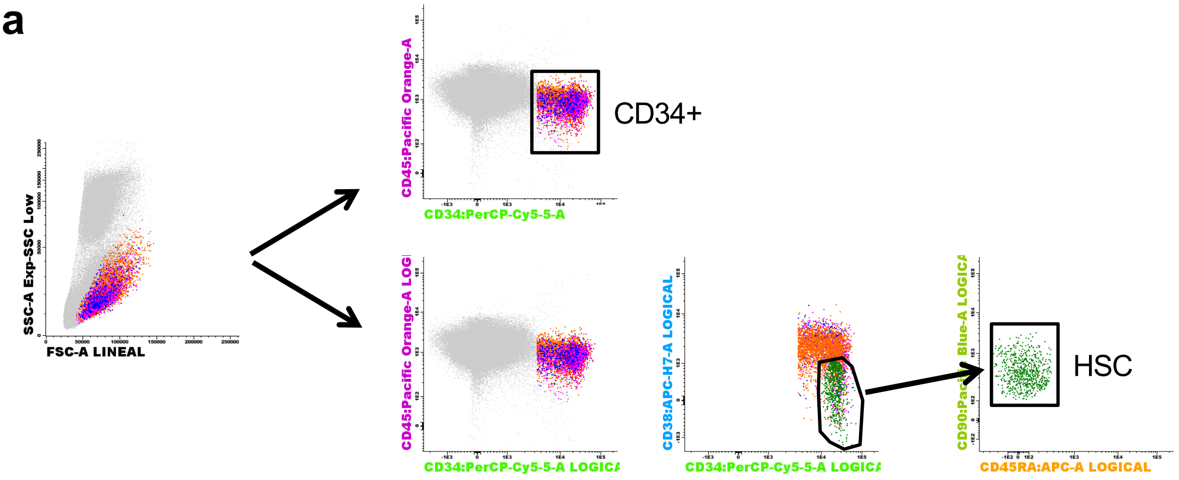
SSC-A Exp-SSC Low
FSC-A LINEAL
CD45:Pacific Orange-A
CD34:PerCP-Cy5-5-A
CD34+
CD34:PerCP-Cy5-5-A LOGICA
CD38:APC-H7-A LOGICAL
HSC
CD45RA:APC-A LOGICAL

b

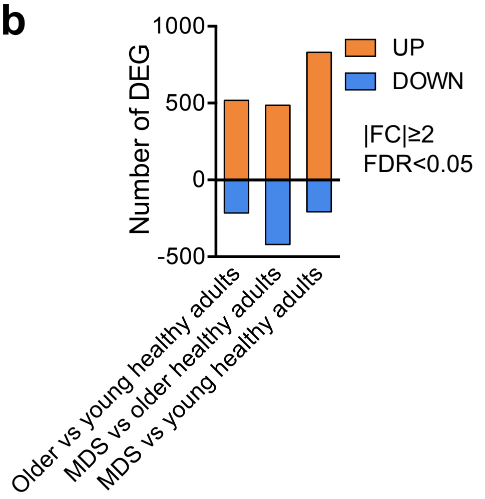
Number of DEG
1000
500
0
-500
UP
DOWN
|FC|≥2
FDR<0.05
Older vs young healthy adults
MDS vs older healthy adults
MDS vs young healthy adults

Figure S2

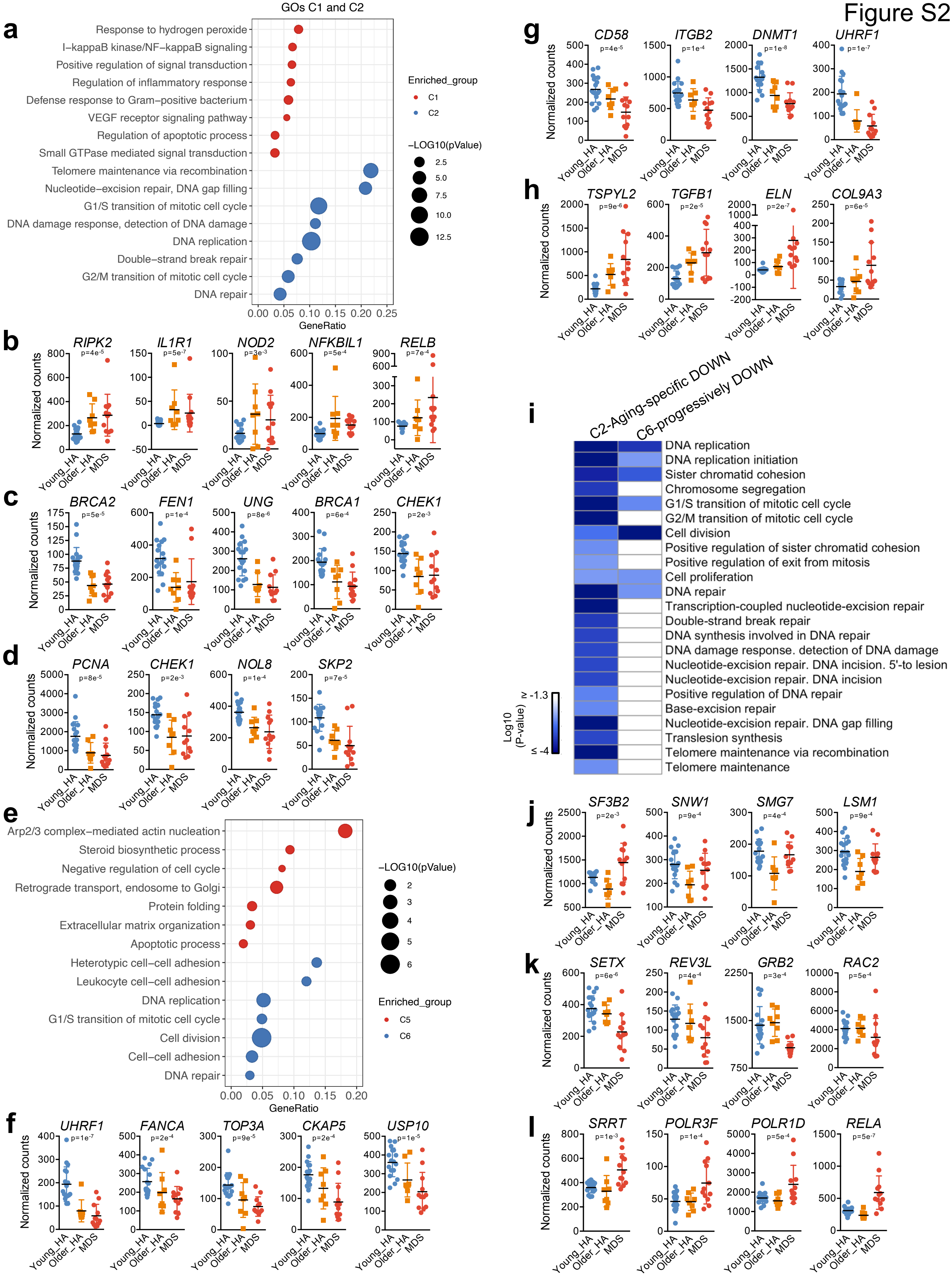

a
GOs C1 and C2
Response to hydrogen peroxide
I−kappaB kinase/NF−kappaB signaling
Positive regulation of signal transduction
Regulation of inflammatory response
Defense response to Gram−positive bacterium
VEGF receptor signaling pathway
Regulation of apoptotic process
Small GTPase mediated signal transduction
Telomere maintenance via recombination
Nucleotide−excision repair, DNA gap filling
G1/S transition of mitotic cell cycle
DNA damage response, detection of DNA damage
DNA replication
Double−strand break repair
G2/M transition of mitotic cell cycle
DNA repair
GeneRatio
Enriched_group
C1
C2
−LOG10(pValue)
b
RIPK2
IL1R1
NOD2
NFKBIL1
RELB
c
BRCA2
FEN1
UNG
BRCA1
CHEK1
d
PCNA
CHEK1
NOL8
SKP2
e
Arp2/3 complex−mediated actin nucleation
Steroid biosynthetic process
Negative regulation of cell cycle
Retrograde transport, endosome to Golgi
Protein folding
Extracellular matrix organization
Apoptotic process
Heterotypic cell−cell adhesion
Leukocyte cell−cell adhesion
DNA replication
G1/S transition of mitotic cell cycle
Cell division
Cell−cell adhesion
DNA repair
GeneRatio
−LOG10(pValue)
Enriched_group
C5
C6
f
UHRF1
FANCA
TOP3A
CKAP5
USP10
g
CD58
ITGB2
DNMT1
UHRF1
h
TSPYL2
TGFB1
ELN
COL9A3
i
C2-Aging-specific DOWN
C6-progressively DOWN
DNA replication
DNA replication initiation
Sister chromatid cohesion
Chromosome segregation
G1/S transition of mitotic cell cycle
G2/M transition of mitotic cell cycle
Cell division
Positive regulation of sister chromatid cohesion
Positive regulation of exit from mitosis
Cell proliferation
DNA repair
Transcription-coupled nucleotide-excision repair
Double-strand break repair
DNA synthesis involved in DNA repair
DNA damage response. detection of DNA damage
Nucleotide-excision repair. DNA incision. 5'-to lesion
Nucleotide-excision repair. DNA incision
Positive regulation of DNA repair
Base-excision repair
Nucleotide-excision repair. DNA gap filling
Translesion synthesis
Telomere maintenance via recombination
Telomere maintenance
Log10 (P-value)
≥ -1.3
≤ -4
j
SF3B2
SNW1
SMG7
LSM1
k
SETX
REV3L
GRB2
RAC2
l
SRRT
POLR3F
POLR1D
RELA
Normalized counts
Young_HA
Older_HA
MDS

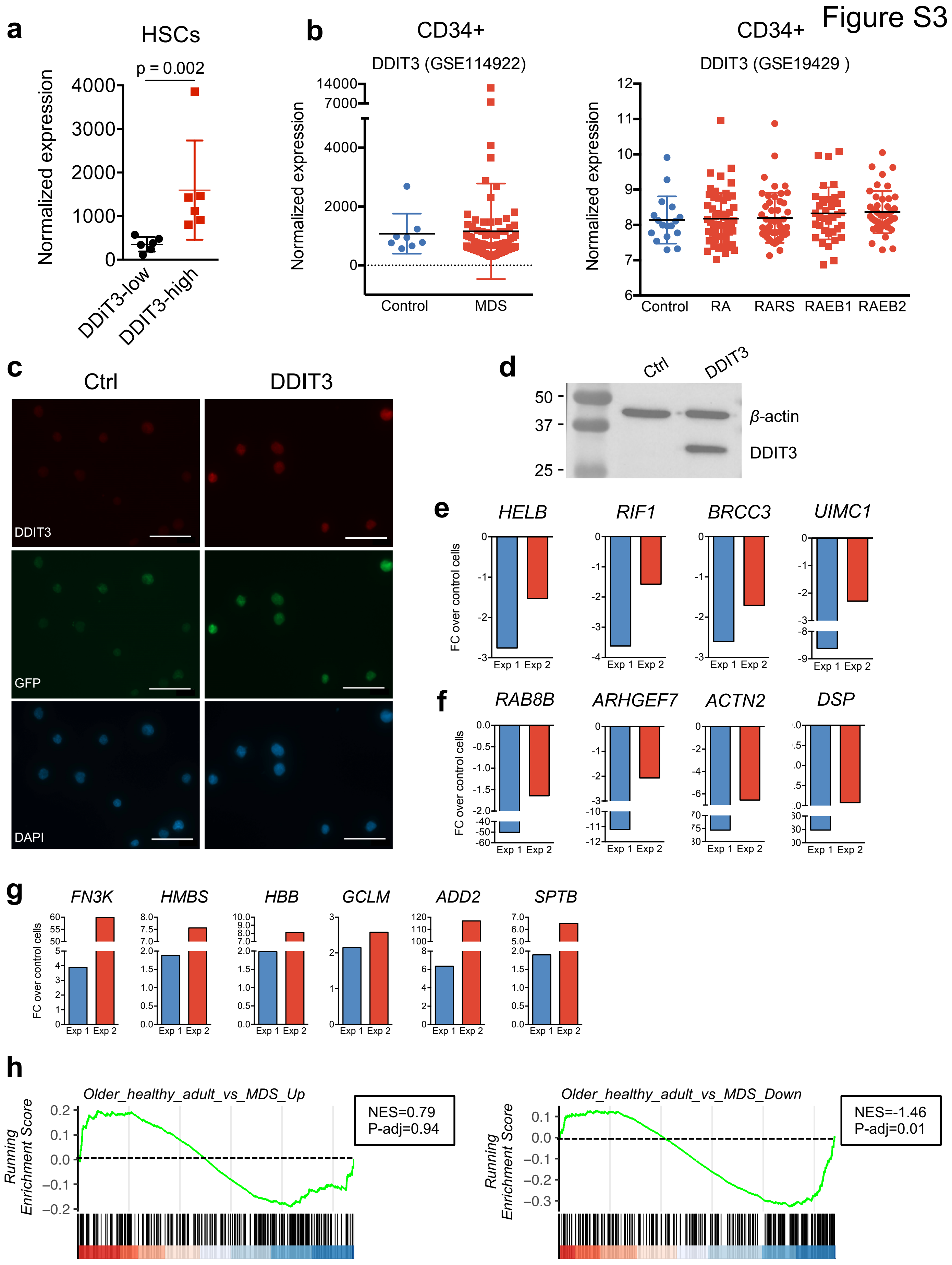
Figure S3
a
HSCs
p = 0.002
Normalized expression
DDiT3-low
DDIT3-high
b
CD34+
DDIT3 (GSE114922)
Normalized expression
Control
MDS
CD34+
DDIT3 (GSE19429)
Normalized expression
Control
RA
RARS
RAEB1
RAEB2
c
Ctrl
DDIT3
DDIT3
GFP
DAPI
d
Ctrl
DDIT3
β-actin
DDIT3
e
HELB
RIF1
BRCC3
UIMC1
FC over control cells
Exp 1 Exp 2
f
RAB8B
ARHGEF7
ACTN2
DSP
FC over control cells
Exp 1 Exp 2
g
FN3K
HMBS
HBB
GCLM
ADD2
SPTB
FC over control cells
Exp 1 Exp 2
h
Older_healthy_adult_vs_MDS_Up
NES=0.79
P-adj=0.94
Running Enrichment Score
Older_healthy_adult_vs_MDS_Down
NES=-1.46
P-adj=0.01
Running Enrichment Score

Figure S4

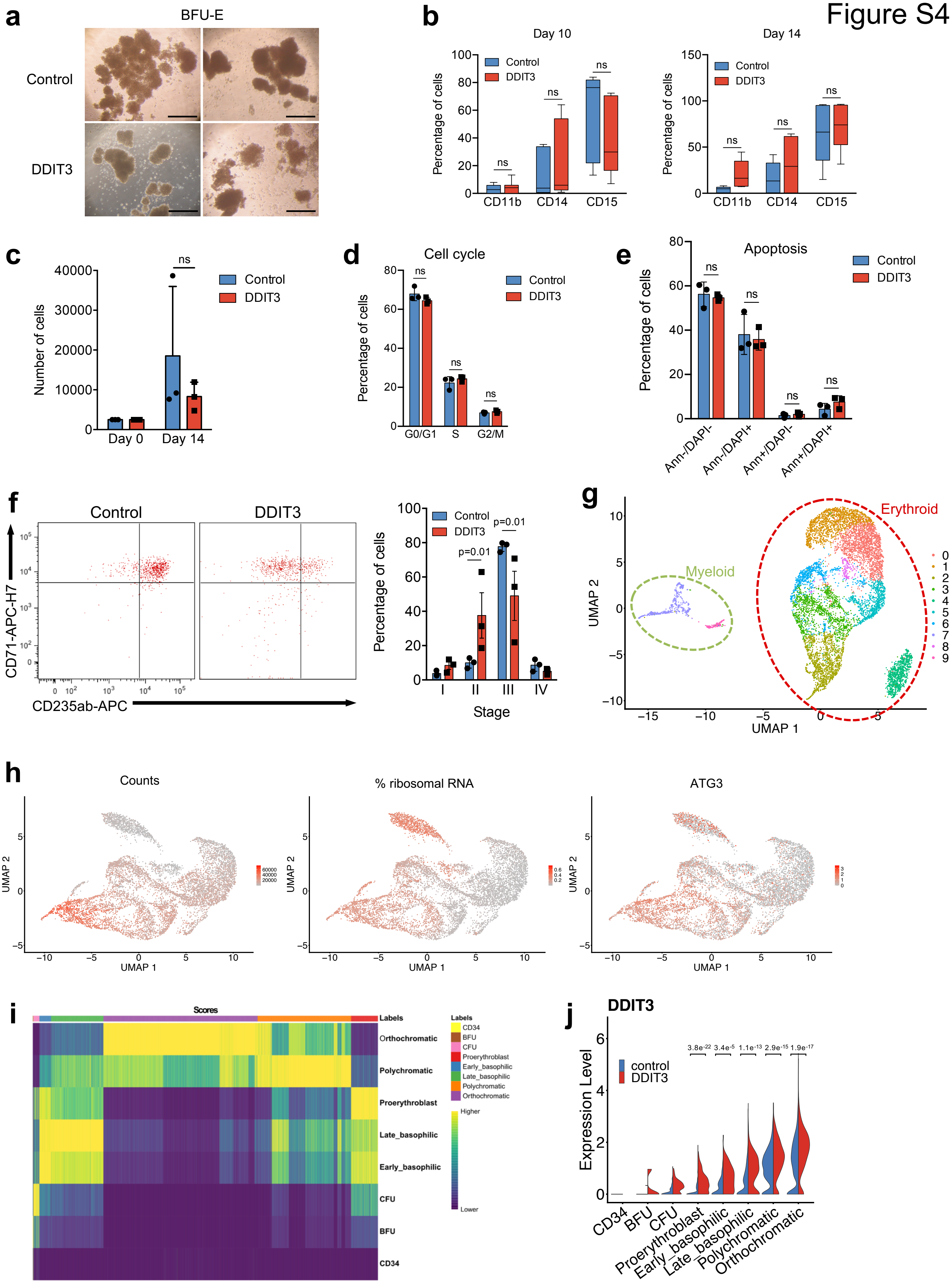

a
BFU-E
Control
DDIT3
b
Day 10
Day 14
Control
DDIT3
Percentage of cells
ns
CD11b
CD14
CD15
c
Number of cells
Day 0
Day 14
d
Cell cycle
G0/G1
S
G2/M
e
Apoptosis
Ann-/DAPI-
Ann-/DAPI+
Ann+/DAPI-
Ann+/DAPI+
f
CD71-APC-H7
CD235ab-APC
p=0.01
Stage
I
II
III
IV
g
Erythroid
Myeloid
UMAP 1
UMAP 2
h
Counts
% ribosomal RNA
ATG3
i
Scores
Labels
Orthochromatic
Polychromatic
Proerythroblast
Late_basophilic
Early_basophilic
CFU
BFU
CD34
Higher
Lower
j
DDIT3
Expression Level
control
DDIT3
3.8e-22
3.4e-5
1.1e-13
2.9e-15
1.9e-17

Figure S5

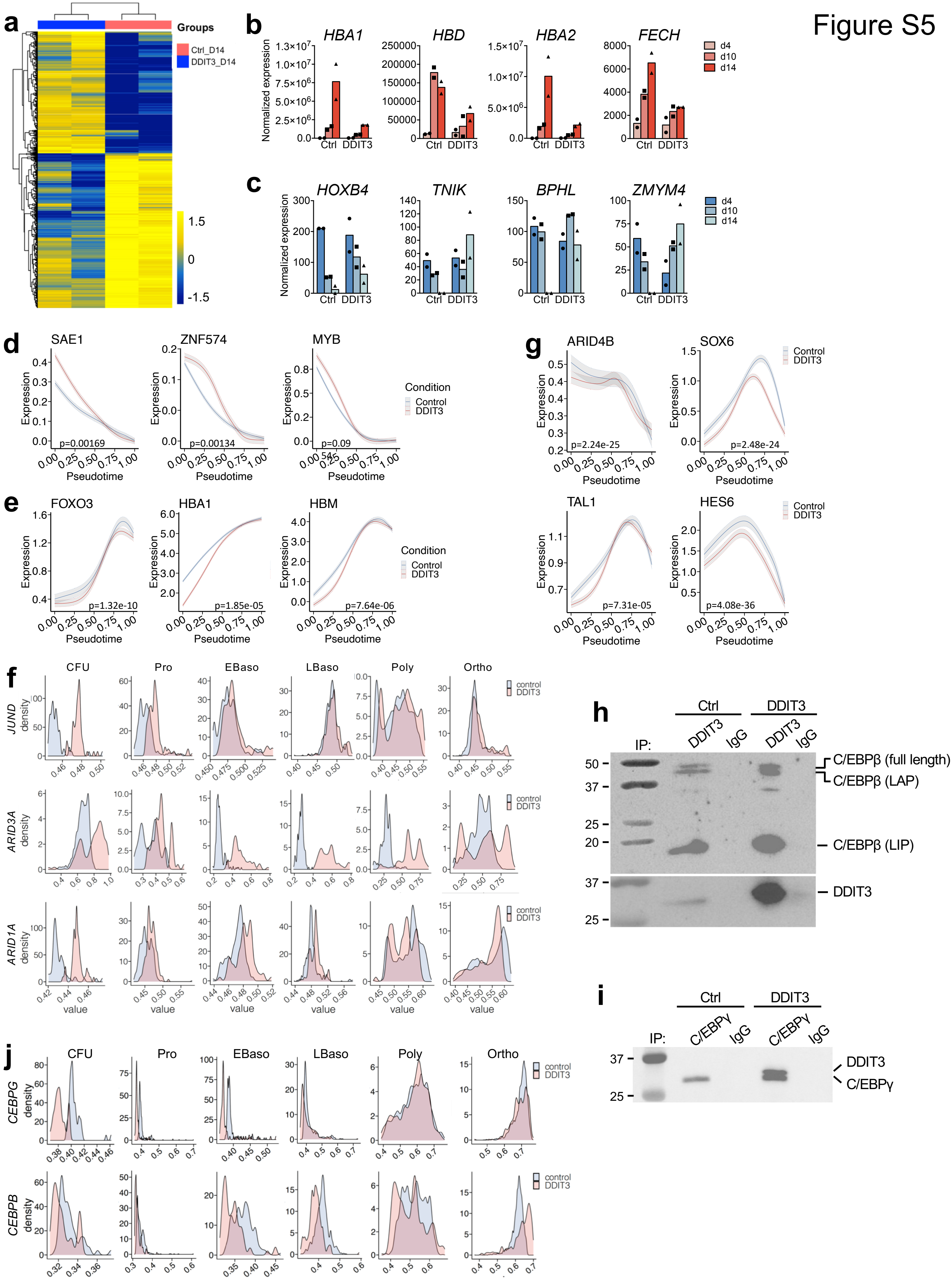

a
Groups
Ctrl_D14
DDIT3_D14
1.5
0
-1.5
b
HBA1
HBD
HBA2
FECH
Normalized expression
Ctrl
DDIT3
d4
d10
d14
c
HOXB4
TNIK
BPHL
ZMYM4
d
SAE1
ZNF574
MYB
p=0.00169
p=0.00134
p=0.09
Condition
Control
DDIT3
Expression
Pseudotime
e
FOXO3
HBA1
HBM
p=1.32e-10
p=1.85e-05
p=7.64e-06
g
ARID4B
SOX6
p=2.24e-25
p=2.48e-24
TAL1
HES6
p=7.31e-05
p=4.08e-36
f
CFU
Pro
EBaso
LBaso
Poly
Ortho
JUND
ARID3A
ARID1A
density
value
control
DDIT3
h
IP:
Ctrl
DDIT3
IgG
C/EBPβ (full length)
C/EBPβ (LAP)
C/EBPβ (LIP)
DDIT3
i
C/EBPγ
j
CEBPG
CEBPB

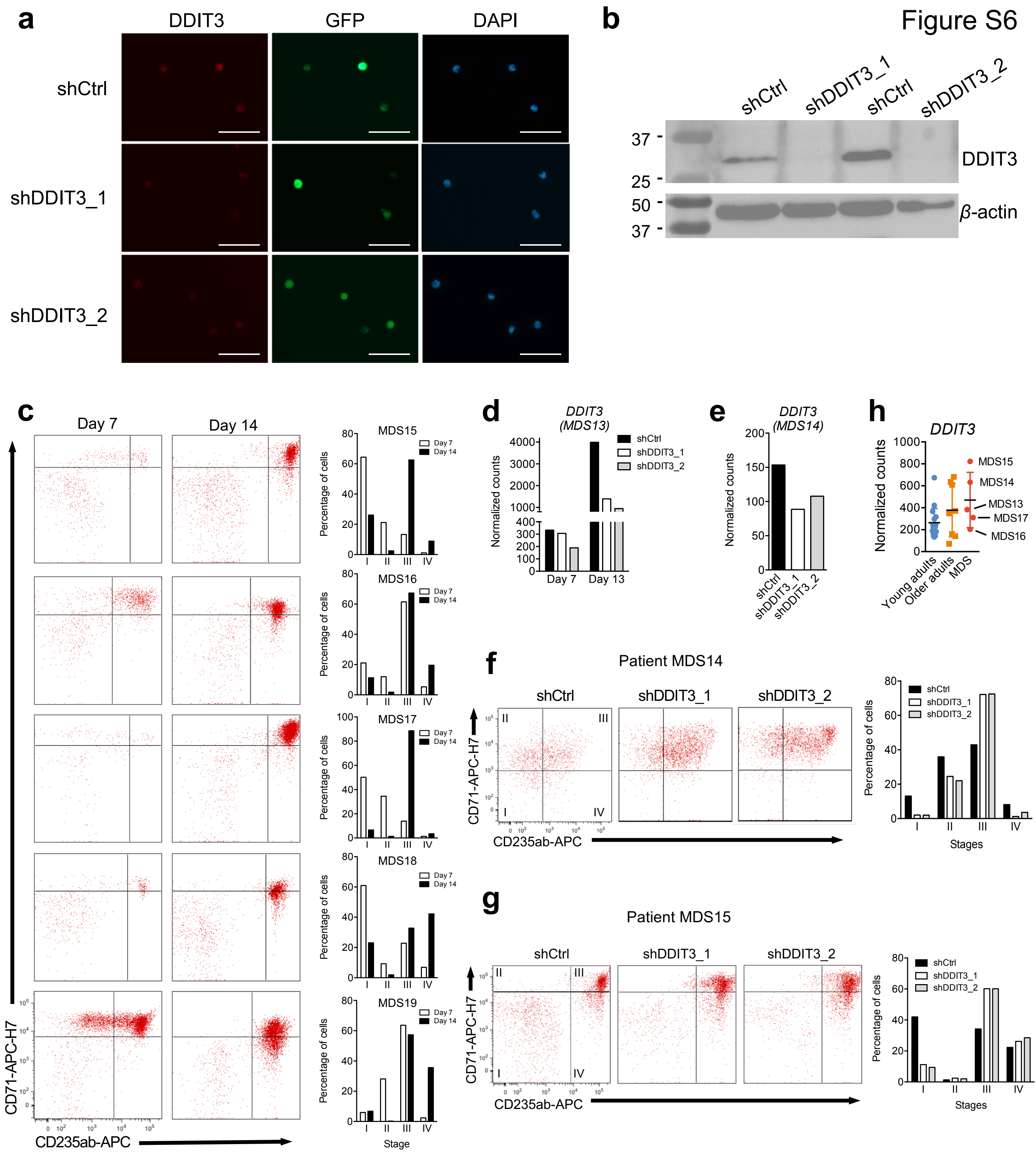
Figure S6
a
DDIT3
GFP
DAPI
shCtrl
shDDIT3_1
shDDIT3_2
b
shCtrl
shDDIT3_1
shCtrl
shDDIT3_2
37
25
50
37
DDIT3
β-actin
c
Day 7
Day 14
CD71-APC-H7
CD235ab-APC
MDS15
MDS16
MDS17
MDS18
MDS19
Day 7
Day 14
Percentage of cells
Stage
d
DDIT3 (MDS13)
Normalized counts
shCtrl
shDDIT3_1
shDDIT3_2
Day 7
Day 13
e
DDIT3 (MDS14)
Normalized counts
shCtrl
shDDIT3_1
shDDIT3_2
h
DDIT3
Normalized counts
Young adults
Older adults
MDS
MDS15
MDS14
MDS13
MDS17
MDS16
f
Patient MDS14
shCtrl
shDDIT3_1
shDDIT3_2
CD71-APC-H7
CD235ab-APC
Percentage of cells
Stages
g
Patient MDS15
shCtrl
shDDIT3_1
shDDIT3_2
CD71-APC-H7
CD235ab-APC
Percentage of cells
Stages

Figure S7

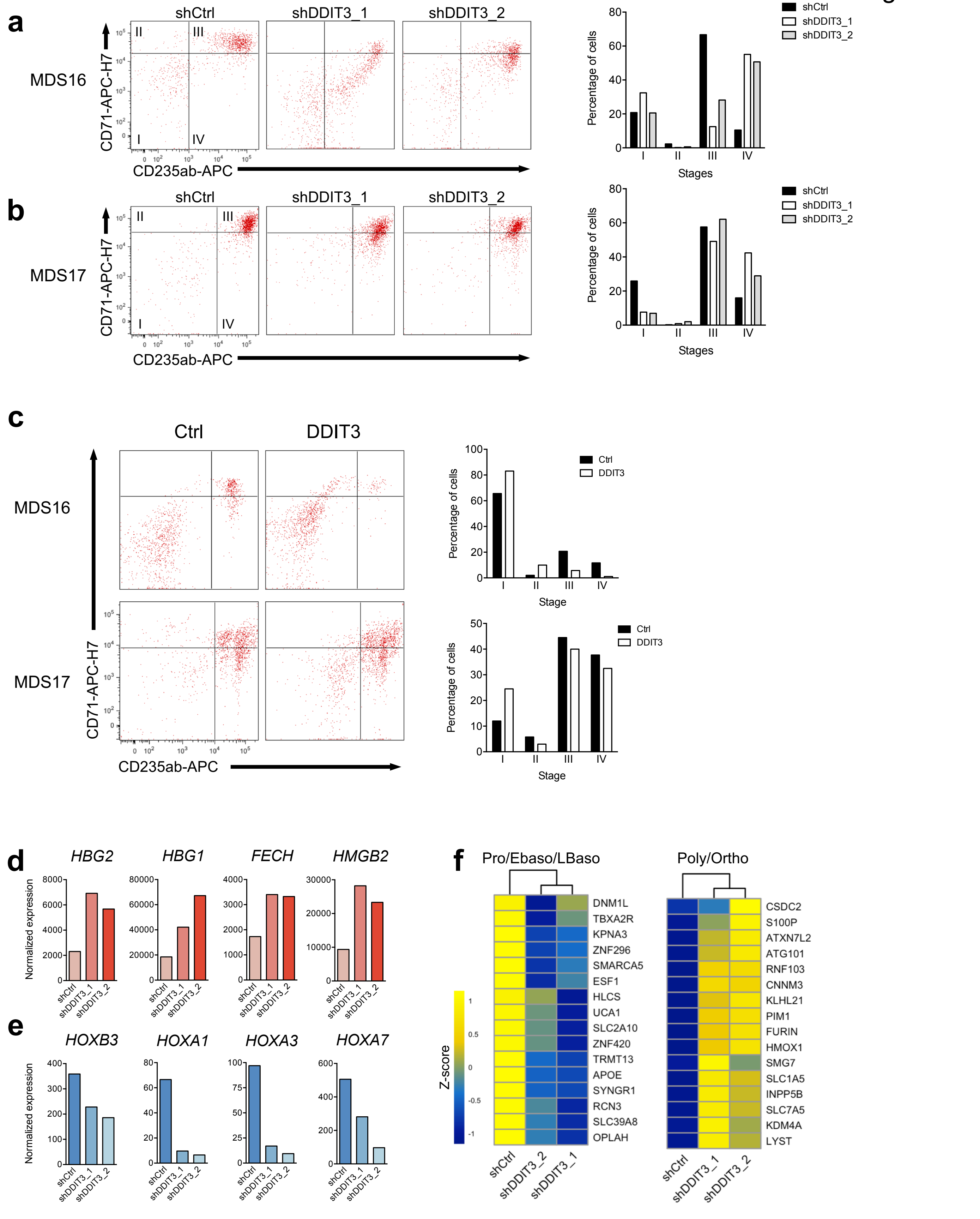

a
MDS16
shCtrl
shDDIT3_1
shDDIT3_2
CD71-APC-H7
CD235ab-APC
I
II
III
IV
Percentage of cells
Stages
b
MDS17
c
Ctrl
DDIT3
Stage
d
HBG2
HBG1
FECH
HMGB2
Normalized expression
e
HOXB3
HOXA1
HOXA3
HOXA7
f
Pro/Ebaso/LBaso
Poly/Ortho
Z-score
DNM1L
TBXA2R
KPNA3
ZNF296
SMARCA5
ESF1
HLCS
UCA1
SLC2A10
ZNF420
TRMT13
APOE
SYNGR1
RCN3
SLC39A8
OPLAH
CSDC2
S100P
ATXN7L2
ATG101
RNF103
CNNM3
KLHL21
PIM1
FURIN
HMOX1
SMG7
SLC1A5
INPP5B
SLC7A5
KDM4A
LYST

Supplementary Table S1
Characeristics of healthy donors (YHA: young healthy adult, OHA: older healthy adult)

ID	Group	Gender	Age	Mutations (%VAF)
YHA_1	Healthy_young_adult	F	20	N/A
YHA_2	Healthy_young_adult	F	20	N/A
YHA_3	Healthy_young_adult	F	21	N/A
YHA_4	Healthy_young_adult	F	22	N/A
YHA_5	Healthy_young_adult	M	21	N/A
YHA_6	Healthy_young_adult	M	19	N/A
YHA_7	Healthy_young_adult	F	20	N/A
YHA_8	Healthy_young_adult	M	19	N/A
YHA_9	Healthy_young_adult	F	19	N/A
YHA_10	Healthy_young_adult	F	20	N/A
YHA_11	Healthy_young_adult	F	18	N/A
YHA_12	Healthy_young_adult	F	22	N/A
YHA_13	Healthy_young_adult	F	22	N/A
YHA_14	Healthy_young_adult	F	22	N/A
YHA_15	Healthy_young_adult	F	22	N/A
YHA_16	Healthy_young_adult	F	20	N/A
YHA_17	Healthy_young_adult	F	22	N/A
YHA_18	Healthy_young_adult	M	22	N/A
YHA_19	Healthy_young_adult	F	22	N/A
YHA_20	Healthy_young_adult	M	20	N/A
YHA_21	Healthy_young_adult	M	20	N/A
YHA_22	Healthy_young_adult	F	23	N/A
YHA_23	Healthy_young_adult	F	20	N/A
YHA_24	Healthy_young_adult	F	22	N/A
YHA_25	Healthy_young_adult	M	21	N/A
YHA_26	Healthy_young_adult	F	20	N/A
YHA_27	Healthy_young_adult	F	20	N/A
YHA_28	Healthy_young_adult	F	21	N/A
YHA_29	Healthy_young_adult	M	20	N/A
YHA_30	Healthy_young_adult	F	22	N/A
OHA_1	Healthy_older_adult	M	64	Not performed
OHA_2	Healthy_older_adult	M	67	Not performed
OHA_3	Healthy_older_adult	F	60	Not performed
OHA_4	Healthy_older_adult	M	58	No mutations
OHA_5	Healthy_older_adult	F	79	No mutations
OHA_6	Healthy_older_adult	F	81	No mutations
OHA_7	Healthy_older_adult	M	58	No mutations
OHA_8	Healthy_older_adult	F	73	DNMT3A (3.1%)

Supplementary Table S2: Clinical characteristics of MDS patients

Sample ID	Gender	Age (years)	Type of MDS (WHO classification)	Treatment	Karyotype	IPSS_R	Mutations (%VAF)
MDS_1	F	86	MDS-MLD	Non-treated	46,XX[30]	Low	No clinically relevant mutations detected
MDS_2	F	60	MDS-MLD	Non-treated	46,XX[20]	Very low	No clinically relevant mutations detected
MDS_3	F	51	MDS-MLD	Non-treated	46,XX[20]	Low	GATA2 (47.83%), JAK2 (43.63%), BCOR (40.61%)
MDS_4	M	87	MDS-MLD	Non-treated	46,XY[20]	Low	ZRSR2 (78%), ASXL1 (40%)
MDS_5	M	87	MDS-MLD	Non-treated	46,XY[20]	Very low	No clinically relevant mutations detected
MDS_6	M	70	MDS-MLD	Non-treated	46,XX[20]	Low	No clinically relevant mutations detected
MDS_7	M	84	MDS-MLD	Non-treated	46,XX[20]	Low	U2AF1 (27.2%), PRPF8 (2.6%)
MDS_8	F	52	MDS-SLD	Non-treated	46, XX [20]	Low	ET2 (44%), IDH2 (42%), U2AF1 (41%), ASXL1 (36%), PHF6 (27%), CBL (6%)
MDS_9	M	70	MDS-MLD	Non-treated	46,XY[20]	Low	TET2 (46.6%), SRSF2 (23.3%), ZRSR2 (19.5%)
MDS_10	M	71	MDS-MLD	Non-treated	46,XY,del(20)(q12)[28/30]/46,XY[2/30]	Low	ZRSR2 (44%), CUX1 (24%)
MDS_11	M	64	MDS-MLD	Non-treated	46,XY[30]	Low	ZRSR2 (97%), TET2 (92%), ASXL1 (46%), KIT (6%)
MDS_12	M	45	MDS-SLD	Non-treated	46,XY[30]	Low	No clinically relevant mutations detected
MDS_13	M	70	MDS-MLD	Non-treated	46,XY[20]	Very low	No clinically relevant mutations detected
MDS_14	M	70	MDS-MLD	Non-treated	46,XY[20]	Low	TET2 (2.6%)
MDS_15	M	77	MDS-MLD	Non-treated	46,XY[21]	Low	DNMT3A (6.3%), PPM1D (3%), SF3B1 (3%), NF1 (4%)
MDS_16	M	74	MDS-MLD	Non-treated	46,XY, del(7)(q22q34)[28]/46,XY[2]	Low	EZH2 (77%), RUNX1 (44%), ASXL1 (38%)
MDS_17	M	84	MDS-EB2	Non-treated	No mitosis (50% del(5q) + 13% del(7q) detected by FISH)	Very high	TP53 (41%), U2AF1 (18%)
MDS_18	F	59	MDS-EB1	Non-treated	45,XX,-7[15]/46,XX[5]	Intermediate	Not performed
MDS_19	M	59	MDS-EB2	Non-treated	46,XY[20]	Intermediate	Not performed

Supplementary Table S3: List of antibodies used for flow cytometry analyses

Antibody	Fluorochrome conjugation	Supplier	Clone	Reference	Lot number	Dilution
CD3	BV510	Biolegend	OKT3	317332	B340677	3:100
CD10	BV510	Biolegend	HI10a	312220	B253991	3:100
CD19	BV510	Biolegend	SJ25C1	363020	B335341	3:100
CD34	PerCP-Cy5.5	BD Biosciences	8G12	347222	2187277	10:100
CD38	APC-H7	BD Biosciences	HB-7	656646	1238088	3:100
CD45RA	APC	BD Biosciences	Clone HI100	550855	200026	5:100
CD64	BV510	Biolegend	10.1	305028	B289011	3:100
CD90	BV421	BD Biosciences	5E10	562556	9030764	1:100
CD235ab	APC	Biolegend	HIR2	306608	B241167	0.25:100
CD71	APC-H7	BD Biosciences	M-A712	655408	2105775	0.5:100
CD11b	APC	BD Biosciences	ICRF44	RRID:AB_10561676	7144899	1:100
CD14	PE-Cy7	BD Biosciences	61D3	RRID:AB_1582277	B275678	1:100
CD15	BV786	BD Biosciences	W6D3	RRID:AB_ 2740635	B277708	1:100
Annexin-V	PE-Cy7	Biolegend	N/A	640912	B263821	1:100
CD45	V500	BD Biosciences	HI30	560777	2230251	5:100
