# Supplementary material for: The transcription factor DDIT3 is a potential driver of dyserythropoiesis in myelodysplastic syndromes
Source: Nat Commun. 2022 Dec 9;13:7619. doi: 10.1038/s41467-022-35192-7 (PMC9734135; doi:10.1038/s41467-022-35192-7)
Supplement: Supplementary file 3 — Reporting Summary [file 41467_2022_35192_MOESM3_ESM.pdf]

nature portfolio

Corresponding author(s): Teresa Ezponda

Last updated by author(s): 8th November 2022

Reporting Summary

Nature Portfolio wishes to improve the reproducibility of the work that we publish. This form provides structure for consistency and transparency in reporting. For further information on Nature Portfolio policies, see our Editorial Policies and the Editorial Policy Checklist.

Statistics

For all statistical analyses, confirm that the following items are present in the figure legend, table legend, main text, or Methods section.

n/a	Confirmed	
☐	☒	The exact sample size (*n*) for each experimental group/condition, given as a discrete number and unit of measurement
☐	☒	A statement on whether measurements were taken from distinct samples or whether the same sample was measured repeatedly
☐	☒	The statistical test(s) used AND whether they are one- or two-sided<br>*Only common tests should be described solely by name; describe more complex techniques in the Methods section.*
☐	☒	A description of all covariates tested
☐	☒	A description of any assumptions or corrections, such as tests of normality and adjustment for multiple comparisons
☐	☒	A full description of the statistical parameters including central tendency (e.g. means) or other basic estimates (e.g. regression coefficient) AND variation (e.g. standard deviation) or associated estimates of uncertainty (e.g. confidence intervals)
☐	☒	For null hypothesis testing, the test statistic (e.g. *F*, *t*, *r*) with confidence intervals, effect sizes, degrees of freedom and *P* value noted<br>*Give P values as exact values whenever suitable.*
☒	☐	For Bayesian analysis, information on the choice of priors and Markov chain Monte Carlo settings
☒	☐	For hierarchical and complex designs, identification of the appropriate level for tests and full reporting of outcomes
☒	☐	Estimates of effect sizes (e.g. Cohen's *d*, Pearson's *r*), indicating how they were calculated

Our web collection on statistics for biologists contains articles on many of the points above.

Software and code

Policy information about availability of computer code

Data collection	No software was used for data collection.
Data analysis	We used the following publicly available software: bcl2fastq (v2.20), STAR (v2.6), DAVID v6.8 (https://david.ncifcrf.gov), GeneScript, GSEA (https://www.gsea-msigdb.org/gsea/index.jsp), CellRanger (v3.1), velocyto.py (v0.17), SimiC (https://github.com/jianhao2016/SimiC)<br>Python language (v3.6) with the modules scVelo (v0.2.1) and palantir (v0.2.1)<br>R language (v3.5) with the packages RSubread (v1.32.4), Seurat (v3.0.0) and MAST (v1.8.2), R language (v4.0) with the package singleR (v1.2.4)<br>R/Bioconductor (v3.11) with the packages DeSeq2, MaSigPro and EnhancedVolcano. For flow cytometry analyses we used BD FACSDIVA v8 and FlowJo v8.7 software.<br>Bioinformatic code regarding the single-cell RNA-seq analyses performed in this work can be found at https://github.com/mainciburu/DDIT3_2022; https://doi.org/10.5281/zenodo.7299427.

For manuscripts utilizing custom algorithms or software that are central to the research but not yet described in published literature, software must be made available to editors and reviewers. We strongly encourage code deposition in a community repository (e.g. GitHub). See the Nature Portfolio guidelines for submitting code & software for further information.

Data

Policy information about availability of data

All manuscripts must include a data availability statement. This statement should provide the following information, where applicable:

- Accession codes, unique identifiers, or web links for publicly available datasets
- A description of any restrictions on data availability
- For clinical datasets or third party data, please ensure that the statement adheres to our policy

Every data generated for this study are available on GEO (GSE183328): https://www.ncbi.nlm.nih.gov/geo/query/acc.cgi?acc=GSE183328.
Bulk RNAseq data from CD34+ cells, to study DDIT3 expression, were downloaded from GEO, with the following accession codes: GSE114922 (https://www.ncbi.nlm.nih.gov/geo/query/acc.cgi?acc=GSE114922) and GSE19429 (https://www.ncbi.nlm.nih.gov/geo/query/acc.cgi?acc=GSE19429).
To classify scRNA-seq cells, bulk RNAseq data from erythroid differentiation were obtained from GSE107218 (https://www.ncbi.nlm.nih.gov/geo/query/acc.cgi?acc=GSE107218). The GRCh38 assembly of the human genome used is available at NCBI, under the accession code NCBI:GCA_000001405.27.

Field-specific reporting

Please select the one below that is the best fit for your research. If you are not sure, read the appropriate sections before making your selection.

☒ Life sciences ☐ Behavioural & social sciences ☐ Ecological, evolutionary & environmental sciences

For a reference copy of the document with all sections, see nature.com/documents/nr-reporting-summary-flat.pdf

Life sciences study design

All studies must disclose on these points even when the disclosure is negative.

Sample size	The transcriptome of HSCs in aging and MDS was characterized in healthy young (n=17), healthy elderly (n=8) and MDS (n=12) bone marrow samples. Sample size was determined by specimen availability, and not by statistical predetermination. Nevertheless, to reduce the heterogeneity associated with MDS patients, MDS cohort included only low- or very low-risk patients with MDS-MLD and MDS-SLD, excluding cases with del(5q), ring sideroblasts or excess blasts. Sample size used was sufficient to find statistically significant gene expression differences among groups and gene trends in aging and MDS. For DDIT3 overexpression assays, 3 independent experiments using 3 bone marrow samples from distinct healthy donors were performed. Single-cell RNA-seq experiments upon DDIT3 overexpression were performed in one of the replicates due to high cost. DDIT3 knockdown experiments were performed in 5 different MDS patients using two different shRNAs.
Data exclusions	No data were excluded in this study.
Replication	For DDIT3 overexpression assays, 3 independent biological experiments using 3 bone marrow samples from distinct healthy donors were performed. Single-cell RNA-seq experiments upon DDIT3 overexpression were performed in one of the replicates due to high cost. DDIT3 knockdown experiments were performed in 5 different MDS patients using two different shRNAs. The results obtained upon DDIT3 overexpression or knockdown were reproducible in all of the samples analyzed. All attempts at replication were successful for both overexpression and knockdown experiments.
Randomization	Not relevant. This study did not involve experimental grouping.
Blinding	Not relevant. This study did not involve experimental grouping.

Reporting for specific materials, systems and methods

We require information from authors about some types of materials, experimental systems and methods used in many studies. Here, indicate whether each material, system or method listed is relevant to your study. If you are not sure if a list item applies to your research, read the appropriate section before selecting a response.

Materials & experimental systems

n/a	Involved in the study	
☐	☒	Antibodies
☐	☒	Eukaryotic cell lines
☒	☐	Palaeontology and archaeology
☒	☐	Animals and other organisms
☐	☒	Human research participants
☒	☐	Clinical data
☒	☐	Dual use research of concern

Methods

n/a	Involved in the study	
☒	☐	ChIP-seq
☐	☒	Flow cytometry
☒	☐	MRI-based neuroimaging

Antibodies

Antibodies used

Anti-CD3 (Biologend, CAT: 317332, Clone: OKT3, Lot number: B340677), anti-CD10 (Biolegend, CAT: 312220, Clone: HI10a, Lot number: B253991), anti-CD19 (Biolegend, CAT: 363020, Clone: SJ25C1, Lot number: B335341), anti-CD34 (BD Biosciences, CAT: 347222, Clone: 8G12, Lot number: 2187277), anti-CD38 (BD Biosciences, CAT: 656646, Clone: HB-7, Lot number: 1238088), anti-CD45RA (BD Biosciences, CAT: 550855, Clone: HI100, Lot number: 200026), anti-CD64 (Biolegend, CAT: 305028, Clone: 10.1 Lot number: B289011), anti-CD90 (BD Biosciences, CAT: 562556, Clone: 5E10, Lot number: 9030764), anti-CD71 (BD Biosciences, CAT: 655408, Clone: M-A712, Lot number: 2105775), anti-CD235a (Biolegend, CAT: 306608, Clone: HIR2, Lot number: B241167), anti-CD11b (BD Biosciences, CAT: RRID, AB_10561676, Clone: ICRF44, Lot number: 7144899), anti-CD14 (BD Biosciences, CAT: RRID, AB_1582277, Clone: 61D3, Lot number: B275678), anti-CD15 (BD Biosciences, CAT: RRID, AB_2740635, Clone: W6D3, Lot number: B277708), anti-annexin-V (Biolegend, CAT: 640912, Clone: N/A, Lot number: B263821), anti-CD45 (BD Biosciences, CAT: 560777, Clone: HI30, Lot number: 2230251).

Validation

All antibodies used have been validated for their use in flow cytometry of human cells.

Eukaryotic cell lines

Policy information about cell lines

Cell line source(s)

The human HEK293T, MM.1S and K562 cell lines, and the murine stromal cell line OP9 were obtained from the ATCC.

Authentication

Cell lines were authenticated by short tandem repeat profiling.

Mycoplasma contamination

The cell lines used tested negative for Mycoplasma contamination.

Commonly misidentified lines (See ICLAC register)

None

Human research participants

Policy information about studies involving human research participants

Population characteristics

Bone marrow samples were obtained from newly diagnosed patients of MDS (average=70 y/o, range=51-87 y/o). Bone marrow samples from healthy controls were obtained from participants undergoing orthopedic surgery (average=67.5 y/o, range=58-81) or from young volunteers (average=20.53 y/o, range = 18-22 y/o). Characteristics of MDS patients and healthy donors are listed in Supplementary Tables S1 and S2.

Recruitment

MDS patients were recruited by their hematologists at the time of diagnosis. Young healthy adults were volunteers recruited by an announcement posted at the University of Navarra´s website. Older healthy adults were patients undergoing orthopedic surgery that were recruited by their doctors.
We did not detect any bias in the recruitment process.

Ethics oversight

Each sample was obtained after the study was approved by the research ethics committee of University of Navarra, and informed consent of all participating subjects was obtained.

Note that full information on the approval of the study protocol must also be provided in the manuscript.

Flow Cytometry

Plots

Confirm that:

- [x] The axis labels state the marker and fluorochrome used (e.g. CD4-FITC).
- [x] The axis scales are clearly visible. Include numbers along axes only for bottom left plot of group (a 'group' is an analysis of identical markers).
- [x] All plots are contour plots with outliers or pseudocolor plots.
- [x] A numerical value for number of cells or percentage (with statistics) is provided.

Methodology

Sample preparation

For isolation of HSCs or CD34+ cells from human from bone marrow specimens, Ficoll-Paque (GE Healthcare #17-1440-093) density gradient centrifugation was performed to enrich for the mononuclear cell population after prior red blood cells lysis. Cells were stained by incubation with the indicated antibodies for 15 minutes.
For flow cytometry of cells subjected to the ex vivo myeloid differentiation system, cells were collected, washed, and stained by incubation for 15 minutes with the indicated antibodies.

Instrument

BD FACSAriaTM IIu sorter (isolation of cell populations and DDIT3 knockdown experiments), and Fortessa flow cytometer (DDIT3 overexpression experiments).

Software	Samples were analyzed using the BD FACSDiva software and FlowJo.
Cell population abundance	For CD34+ cells, the purity was determined by re-analyzing part of the sorted population by flow cytometry, obtaining >98% CD34+ cells.<br>The purity of GFP+ sorted population was checked using a Nexcelom cell counter, which allows for the detection of green fluorescent cells. We detected >99% of purity due to restrictive gating conditions used.
Gating strategy	For isolation of HSCs, cells were gated by size (FSC/SSC). Cells positive for CD34, and negative for CD38 were selected. Within that population, cells that expressed CD90 but negative for CD45RA were sorted as HSCs. An example of the gating strategy is shown in figure S1A.<br>For isolation of CD34+ cells, cells were gated by size (FSC/SSC), and sorted based in CD34 and CD45 expression (CD34 positive expression and CD45 medium expression).<br>For DDIT3 overexpression and knockdown experiments, GFP+ cells (transduced cells) were sorted.

☒ Tick this box to confirm that a figure exemplifying the gating strategy is provided in the Supplementary Information.
