# Supplementary material for: The transcription factor DDIT3 is a potential driver of dyserythropoiesis in myelodysplastic syndromes
Source: Nat Commun. 2022 Dec 9;13:7619. doi: 10.1038/s41467-022-35192-7 (PMC9734135; doi:10.1038/s41467-022-35192-7)
Supplement: Supplementary file 4 — Description of Additional Supplementary Files [file 41467_2022_35192_MOESM4_ESM.docx]

**Description of Additional Supplementary Files**

File Name: Supplementary Data 1

Description: Lists of differentially expressed genes between HSCs from young healthy adults, older healthy adults, and MDS patients.

File Name: Supplementary Data 2

Description: List of genes per cluster.

File Name: Supplementary Data 3

Description: Log10 p-value of processes and pathways enriched in each cluster.
